# Factors associated with using disposable versus non-disposable electronic cigarettes among adults in the U.S. and Israel: a cross-sectional study with policy implications

**DOI:** 10.1186/s13584-025-00738-9

**Published:** 2025-12-03

**Authors:** Begashaw A. Mulu, Carla J. Berg, Hagai Levine, Lorien C. Abroms, Yan Wang, Yael Bar-Zeev

**Affiliations:** 1 Braun School of Public Health and Community Medicine, Faculty of Medicine, Hebrew University of Jerusalem-Hadassah Medical Centre, Jerusalem, Israel; 2 Milken Institute School of Public Health, George Washington University, Washington, DC USA

## Abstract

**Background:**

In recent years, there has been a rapid increase in disposable electronic cigarette (e-cigarette) use. Several countries have banned or are considering banning disposable e-cigarettes, which might affect adults who use disposable e-cigarettes for harm reduction. Understanding patterns of disposable versus non-disposable e-cigarette use among adults who currently use e-cigarettes is essential to inform possible regulations and policy. This study explored factors associated with disposable versus non-disposable e-cigarette use among adults currently using e-cigarettes in the U.S. and Israel.

**Method:**

A cross-sectional online survey was conducted (October-December 2021) among 410 U.S. and Israeli adults aged 18–45 years (mean age 30.8 ± 7.6) who reported past 30-day e-cigarette use (62.9% of whom used non-disposables). A multivariate logistic regression assessed factors associated with disposable versus non-disposable e-cigarette use, stratified by country.

**Result:**

In Israel, a greater perceived harm of e-cigarettes (aOR = 1.45, 95%CI: 1.20–1.76), cannabis/cannabinoid-containing e-liquid use ( aOR 0.36, 95%CI: 0.19–0.69) and purchasing from ‘regular’ shops (aOR = 6.30, 95%CI: 2.25–17.67) or ‘specialty’ shops (aOR = 3.71, 95%CI: 1.21–11.34) (compared to ‘online’ shops) were associated with disposable e-cigarette use. In the U.S., factors associated with disposable e-cigarette use included ever using other tobacco/nicotine products (aOR = 7.51, 95%CI: 1.49–37.87), sweet flavor preference (aOR = 4.42, 95%CI: 1.64–11.94), cannabis/cannabinoid-containing e-liquid use (aOR = 0.33, 95% CI: 0.13–0.82), and being 26–35 years old (vs. 36–45) (aOR = 0.36, 95% CI: 0.13–0.98).

**Conclusions:**

U.S. and Israeli adults who use disposable versus non-disposable e-cigarettes differ, highlighting the importance of public health strategies tailored to the unique needs of each country. The high levels of cannabis or cannabinoid-containing e-liquid use among participants who use non-disposable e-cigarettes suggest the need for stricter regulation and enforcement of non-disposable devices and e-liquids in both countries. Findings also suggest that banning sweet flavored e-cigarettes will support the effort in reduction of disposable e-cigarette use in the U.S.

**Supplementary Information:**

The online version contains supplementary material available at 10.1186/s13584-025-00738-9.

## Background

Electronic cigarettes (e-cigarettes) are battery-powered devices that heat a liquid to produce an inhalable aerosol containing nicotine, flavors, propylene glycol, and vegetable glycerin [1, 2]. The e-cigarette market offers a range of products, including disposable, refillable, and pod-based systems [1]. Disposable e-cigarettes, designed for single use, are pre-filled with e-liquid and powered by non-rechargeable batteries [3–5]. These devices often contain higher nicotine strengths (16–20 mg), delivering a smoother experience via nicotine salts [3–5]. Non-disposable e-cigarettes are customizable, featuring rechargeable batteries and refillable tanks [3–5]. These devices come in various styles, including pre-filled pods, mods, and starter kits [3–5].

While there is a scientific consensus that e-cigarette use is harmful and highly addictive, the exact health risks associated with e-cigarettes are still not fully known [6]. Most experts agree that they offer a less harmful alternative for adults who smoke and are not able to quit, as they do not involve combustion [7]. However, concerns about long-term risks, including cardiovascular and lung disease, nicotine addiction, and youth uptake, persist [8–11]. Additionally, malfunctioning devices pose safety hazards, and disposable e-cigarettes contribute significantly to environmental pollution and electronic waste [8, 12, 13].

In the U.S., 6.5% of adults reported using e-cigarettes in 2023, up from 4.5% in 2021 [14]. This rise is primarily driven by young adults aged 18–24, among whom e-cigarettes have overtaken combustible cigarettes in popularity [14, 15]. Among adults who use e-cigarettes, 66% of those aged 18–24 had never smoked cigarettes, compared to 21.6% of those aged 25–44 and 11.3% of those aged 45–64 [14]. Disposable e-cigarette sales have also increased significantly, representing 19.8% of total e-cigarette sales between 2019 and 2020, while prefilled cartridge sales dropped [16]. In 2022, e-cigarettes were the most popular nicotine product among U.S. high school (14.1%) and middle school students (3.3%) [17]. Among those who reported using e-cigarettes, 53.7% reported using disposable devices [17].

In Israel, in a representative sample of adults in 2021, 1.6% reported using e-cigarettes, with higher prevalence among the Arab population (1.8%) compared to the Jewish population (1.2%) and among men (2.9%) compared to women (0.4%) [18]. Repeated cross-sectional panel surveys show that among those aged 18–24, e-cigarette use has tripled from 3.8% in 2020 to 10.1% in 2022 [19]. The most significant increase in adult use of disposable e-cigarettes was observed among the younger age group of 18 to 24 [18, 19]. In contrast, the consumption of refillable e-cigarettes did not show age dependency, being slightly more common among individuals aged 18 to 29, and only slightly less frequent among those aged 30 to 64 [18, 19].

Countries vary widely in their regulatory approaches to e-cigarettes from no regulation to prohibition [20, 21]. In the United States (U.S.), e-cigarettes are subject to pre-market authorization, meaning that manufacturers must obtain approval from the Food and Drug Administration (FDA) before marketing their products [22]. The U.S. also allows branded packaging, although all e-cigarette packaging is required to display health warnings, covering 30% of the package, stating: ‘’Warnings: This product contains nicotine. Nicotine is an addictive chemical.’’ (Supplemental file 1) [22]. Sales of e-cigarettes are restricted to individuals over the age of 21, in line with federal regulations [22]. However, while there are health-related advertising restrictions, there are currently no specific measures that regulate the advertising or display of e-cigarettes in public spaces [22]. There is no federal tax on e-cigarettes, although several states have implemented their own specific taxes on these products [23].

In Israel, e-cigarettes do not require pre-market authorization, meaning manufacturers can sell these products without prior approval from the government [24]. The country enforces plain packaging regulations, which prohibit the use of branded packaging for e-cigarette products [24]. Health warnings must also appear on 30% of the packaging, though their content differs from those required in the U.S., with a statement that reads: “Warning: This Product is highly addictive and harmful to your health” (Supplemental file 1) [24]. In terms of age restrictions, e-cigarettes can be sold to individuals over the age of 18. Israel has stricter bans on advertising and display of e-cigarettes, making these less common compared to the U.S [24]. In Israel, a specific tax is imposed on e-cigarettes, similar to the tax applied to cigarettes [19].

Currently, neither the U.S. nor Israel has specific restrictions on disposable e-cigarettes [22–24]. Some countries, such as the UK, Netherlands, and France, have either banned or are considering banning disposable e-cigarettes due to rising youth use and associated health risks [25–27]. However, concerns have been raised that banning disposable e-cigarettes might impact harm-reduction options for adults who smoke and are unable to quit, making it harder to switch to e-cigarettes [28].

A few studies have examined factors associated with the use of disposable versus non-disposable e-cigarettes among youth and young adults [12, 29–31]. These studies found that factors such as older age (17–18 years versus 9–14 years), non-Hispanic Black ethnicity, nicotine-containing and flavored e-cigarette use, prior vaping experience, preference for ice flavors, low perceived harm of e-cigarettes, greater exposure to e-cigarette marketing, identifying as female or LGBTQ, and perceptions of disposables as more attractive, modern, and appealing in flavor and design were all associated with increased odds of disposable e-cigarette use [12, 29–31]. However, no studies to date have examined these factors among adults currently using e-cigarettes. Understanding the pattern of use of disposable versus non-disposable e-cigarettes among adults who use e-cigarettes is essential to inform possible regulations and policy. This study aims to explore factors associated with using disposable e-cigarettes compared to non-disposable alternatives among adults in both the U.S. and Israel.

## Methods

### Study design and data source

This study is a secondary data analysis from a cross-sectional online survey conducted by Ipsos [32] from October to December 2021 in the U.S. and Israel [33]. The survey was part of a broader study aiming to explore factors associated with using heated tobacco products. Participants were eligible if they were citizens of the respective countries, aged 18–45, and fluent in English (for U.S. participants) or Hebrew/Arabic (for Israeli participants) [33]. The final sample included 2,222 participants, with 1,128 from the U.S. and 1,094 from Israel [33]. The study over-sampled adults reporting tobacco use (by 40%) to ensure sufficient representation of this subgroup [33]. The U.S. sample was primarily drawn from Knowledge Panel^®^ (KP), a probability-based web panel designed to be representative of the national population, recruited using random digit dialing and address-based sampling. KP members received points redeemable for cash (approximately 5,000 points, equivalent to $5, for a 25-minute survey). As standard with Knowledge Panel^®^ surveys, multiple prompts (i.e., on days 3, 6, 14, 21, 28, and 35) were made to encourage participation. Of 4,960 panelists recruited, 2,397 (48.3%) completed eligibility screening, and 1,095 (45.7%) of those eligible completed the survey [33]. To meet subgroup recruitment targets, Ipsos also collected an opt-in (i.e., off-panel) convenience sample of Asian tobacco users. Individuals were recruited using banner ads, web pages, and e-mail invitations; those who clicked on online ads completed eligibility screening (i.e., gender, race/ethnicity, tobacco use). Of 353 individuals screened and eligible, 33 (9.3%) completed the survey [33]. In Israel, data were collected entirely through an opt-in sample of individuals who signed up to participate in surveys. This was conducted by Ipsos in collaboration with several Israeli panel companies in exchange for compensation, (similar to the US, equivalent to $5). Ipsos used an opt-in sample in Israel to reach Jewish and Arab adults aged 18 to 45 years old, living in Israel with an Israeli identification card. Of 2,970 eligible respondents, Ipsos obtained a total of 1,094 (36.8%) who completed the survey. Full details of the recruitment process can be found elsewhere [33]. The present study included only participants who reported using e-cigarettes in the past 30 days (18.5% of all participants in both countries, *n* = 410).

### Measures

The primary outcome was type of e-cigarette reported being used most frequently, with four options (disposable, a device that uses replaceable pre-filled cartridges, a refillable tank, or a mod system), dichotomized to using disposable e-cigarettes vs. non-disposable e-cigarettes (a device that uses replaceable pre-filled cartridges, a refillable tank, or a mod system).

**Sociodemographic factors:** included age, gender, race/ethnicity, education level, income, employment, relationship status, nativity (defined as birthplace of the respondent, e.g., born in U.S. vs. born outside of U.S. for the U.S. subsample, and born in Israel vs. born outside of Israel for the Israel subsample), sexual orientation, and religious level. 

**Perceptions and attitudes toward e-cigarettes**: Harm perception of e-cigarettes was measured on a 7-point Likert scale, ranging from “Not at all harmful” (1) to “Extremely harmful” (7). Therefore, higher scores reflect a stronger belief that e-cigarettes are harmful, while lower scores indicate a belief that they are less harmful. Similarly, the perceived addictiveness of e-cigarettes was measured on a 7-point Likert scale, where 1 indicated “Not at all addictive” and 7 indicated “Extremely addictive.” To assess the perception of how e-cigarettes compare to combustible cigarettes, a 5-point Likert scale was used, ranging from “Not at all” (1) to “Very” different (5). Therefore, higher scores indicate a stronger belief that e-cigarettes are different from combustible cigarettes, while lower scores suggest participants viewed them as more similar. Respondents’ perception of the information they had received about e-cigarettes was measured on a 5-point Likert scale, ranging from “Mostly positive” (1) to “Mostly negative“ (5). The effect of health warnings on their thoughts on using e-cigarettes was measured using four categories (“Have not seen or noticed”, “Made me concerned”, “Reassured me”, or “Had no effect”). The importance of quitting e-cigarettes and confidence in their ability to quit was measured on a scale from 0 to 10, with higher scores indicating greater importance and confidence, respectively. The likelihood of continued e-cigarette use in the next year was assessed using a 7-point Likert scale from (1) “Not at all likely” to (7) “Extremely likely,” while respondents’ plans to quit in the future were categorized by time frame: “≤1 month,” “>1 month,” and “No plan to quit.” Finally, a dichotomous variable assessed whether respondents had attempted to quit using e-cigarettes in the past year (Yes/No).

**Use of tobacco and other substances:** Ever use and current use (defined as past 30-day use) were assessed for combustible cigarettes separately, and all other tobacco and nicotine products combined (including heated tobacco products, water pipe, cigars, and smokeless tobacco). Current (past 30 day) use of cannabis and alcohol were also assessed with those who used for 0–1 day categorized as “No” and > 1 day as “Yes”.

**E-cigarette use characteristics (frequency, concentration, flavor): **For e-cigarettes, we also assessed daily use (Yes/No) in the past 30 days, and daily puff use, dichotomized to ˂39 puffs or ≥ 40. E-cigarette use upon waking in the morning was categorized as “≤5 minutes” or “>5 minutes” after waking. The use of e-liquids with cannabinoids (CBD) or cannabis in e-cigarettes was also assessed. Nicotine concentration in the e-liquids most frequently used was categorized into three groups: “<5%”, “5.0–5.9.0.9%”, and “>6%.” E-cigarette flavor most frequently used (with the option of selecting up to three flavors) was categorized as “Tobacco-flavored,” “Menthol or mint-flavored”, “Sweet (fruit, caramel, and candy)-flavored”, and “Others (alcohol, coffee, or tea)”.

**Location for purchase of e-cigarette: **The most common places for purchasing vape products, e-cigarettes, and/or e-liquids were categorized as ’Regular’ Shops (including gas stations, convenience stores, grocery stores, pharmacies, and mall kiosks), ’Specialty’ shops (including vape shops, and specialty tobacco stores), ‘Online’ shops (online via a site connected to a local vape shop, online via a vendor not connected to a local vape shop), and ‘Others’ (like liquor stores, duty free shop or military commissary, outside the country).

## Analysis

Descriptive statistics were computed for the entire sample, including frequencies and percentages for categorical variables and means and standard deviations for continuous variables. Bivariate analyses were conducted using Pearson’s Chi-square test for categorical variables such as age, gender, education status, and employment status. Mann-Whitney U test was used for ordinal variables, including perceptions of harm, differences between combustible cigarettes and e-cigarettes, e-cigarette information, perceived e-cigarette addictiveness, importance to quit e-cigarettes, and confidence to quit e-cigarettes, and the likelihood of continuing e-cigarette use in the next year, to examine their association with the primary outcome of e-cigarette type (disposable vs. non-disposable). Moreover, the Mann–Whitney U test was used to compare age as a continuous variable, as it did not follow a normal distribution among those who used disposable e-cigarettes and those who used non-disposable e-cigarettes. For variables with significant differences and three or more categories such as age, annual household income, e-cigarettes purchasing locations, and plan to quit e-cigarettes, a post hoc analysis was applied to identify specific group differences in the variable categories between those using disposable e-cigarettes and those using non-disposable e-cigarettes. Statistical significance was adjusted for multiple hypothesis testing by applying Bonferroni correction. Variables that were significant (*p* < 0.05) in the bivariate analysis and were deemed to be of clinical significance were included in the final multivariate logistic regression model. Gender was included in the model as a universal confounder, regardless of its association. To refine the model, we initially identified 21 significant variables but due to sample size constraints, we then reduced the number of variables by lowering the p-value (from < 0.1 to < 0.05), eliminating highly correlated variables, combining similar variables, and selecting those with clinical significance.

Multicollinearity was assessed using linear regression, which showed acceptable Variance Inflation Factor (VIF) values for all independent variables (VIF < 5), indicating that multicollinearity was not a concern in this analysis. Additionally, the tolerance values were greater than 0.1, further confirming the absence of problematic multicollinearity.

Interaction analysis revealed that the country of residence modifies the association between age, annual household income, and use of other tobacco or nicotine products (excluding cigarettes and e-cigarettes) with the use of disposable versus non-disposable e- cigarettes, all of them with a p-value of < 0.05. The bivariate analysis was repeated and stratified by country for comparison. Since no significant differences were found, we chose to use unstratified bivariant analysis result to get similar variables in our analysis to allow for a comparison of factors associated with those who used disposable e-cigarettes versus those who used non-disposable e-cigarettes. Additionally, the logistic regression analysis was stratified by country (Israel/U.S.), and results were presented as adjusted odds ratios (aOR) with 95% confidence intervals (CI), with a significance level of α = 0.05 for all tests. Goodness of fit of the logistic regression model on Israel data and U.S. data were assessed, which showed both of them as a good fit for the model.

## Results

### Sociodemographic characteristics

Overall, of the *n* = 410 participants, *n* = 152 (37.1%) reported using disposable e-cigarettes and *n* = 258 (62.9%) using non-disposable e-cigarettes. The mean age was 30.8 ± 7.6, 55.4% (*n* = 227) of the respondents were male, 67.6% (*n* = 276) attended college or above, 56.4% (*n* = 229) were married or living with a partner, 65.8% (*n* = 258) were categorized as having a middle level of income, and 72.0% (*n* = 295) of the respondents were employed (Table 1). Those reporting disposable e-cigarette use were primarily aged 18–25 (45.5%, *n* = 69), whereas those reporting non-disposable use had higher proportions in the 26–35 (40.7%, *n* = 105) and 36–45 (35.7%, *n* = 92) categories (*p* < 0.001). While 38.5% of study participants resided in U.S., a larger proportion of participants with disposable versus non-disposable use resided in Israel (69.7%, *n* = 106 vs. 56.6%, *n* = 146).

Stratifying the analysis by country revealed notable sociodemographic differences in disposable e-cigarette use (Table 1). In Israel, nearly half (48.1%, *n* = 51) of adults reporting disposable e-cigarette use were aged 18–25, compared to only 27.6% (*n* = 40) among those reporting non-disposable e-cigarette use (*p* = 0.002). Similarly, in the U.S. 39.1% (*n* = 18) of adults reporting disposable e-cigarette use were aged 18–25, vs. 18.8% (*n* = 21) among adults reporting non-disposable e-cigarette use (*p* = 0.01), indicating greater prevalence among younger adults. Additionally, in Israel, a smaller proportion of adults reporting disposable product use were married or cohabitating compared to those reporting non-disposable use (48.6% (*n* = 51) vs. 70.1% (*n* = 101), *p* < 0.001), with no significant difference found in the U.S. sample.

**Table 1:** Sociodemographic characteristics across the total sample of U.S. And Israeli adults, And stratified by country, across disposable versus non-disposable e-cigarette use (*n* = 410)

	Overall (n = 410)				Israel (n = 252)				U.S. (n = 158)			
Variables	**Total (n = 410)** **n (%)**	**Disposable (n = 152)** **n (%)**	**Non-disposable (n = 258)** **n (%)**	**p-value **	**Total (n = 252)** **n (%)**	**Disposable (n = 106)** **n (%)**	**Non-disposable (n = 146)** **n (%)**	**p-value **	**Total (n = 158)** **n (%)**	**Disposable (n = 46)** **n (%)**	**Non-disposable (n = 112)** **n (%)**	**p-value **
**Age (Mean ± SD)**	30.8 ± 7.6	30.3 ± 7.5	31.1 ± 7.8	0.28ᵃ	30.7 ± 7.7	30.7 ± 7.3	30.8 ± 8.0	0.99ᵃ	30.9 ± 7.6	29.4 ± 7.7	31.6 ± 7.5	0.08ᵃ
**Age**												
18–25	130 (31.7)	69 (45.5)	61 (23.6)	**< 0.001**	91 (36.1)	51 (48.1)	40 (27.6)	0.002	39 (24.7)	18 (39.1)	21 (18.8)	**0.01**
26–35	149 (36.3)	44 (28.9)	105 (40.7)		89 (35.3)	33 (31.1)	56 (38.4)		60 (38.0)	11 (23.9)	49 (43.8)	
36–45	131 (32.0)	39 (25.7)	92 (35.7)		72 (28.6)	22 (20.8)	50 (34.2)		59 (37.3)	17 (37.0)	42 (37.5)	
**Gender**												
Male	227 (55.4)	79 (52.0)	148 (57.4)	0.29	140 (55.6)	54 (50.9)	86 (58.9)	0.21	87 (55.1)	25 (54.3)	62 (55.4)	0.91
**Education level¹**												
≥ College	276 (67.6)	95 (62.9)	181 (70.4)		179 (71.6)	72 (68.6)	107 (73.8)		97 (61.4)	23 (50.0)	74 (66.1)	
**Sexual orientation**												
Heterosexual	329 (80.2)	126 (82.9)	203 (78.7)	0.30	204 (81.0)	85 (80.2)	119 (81.5)	0.79	125 (79.1)	41 (89.1)	84 (75.0)	0.05
**Relationship status¹**												
Married/cohabitating	229 (56.4)	69 (45.7)	160 (62.7)	**< 0.001**	152 (61.0)	51 (48.6)	101 (70.1)	**< 0.001**	77 (49.0)	18 (39.1)	59 (53.2)	0.11
E**mployment status**												
Employed	295 (72.0)	102 (67.1)	193 (74.8)	0.09	183 (72.6)	70 (66.0)	113 (77.4)	**0.04**	112 (70.9)	32 (69.6)	80 (71.4)	0.82
**Annual household income ¹**												0.10
Low Income	81 (20.7)	39 (27.5)	42 (16.8)	**0.04**	47 (20.1)	25 (26.1)	22 (15.9)	0.16	34 (21.5)	14 (30.4)	20 (17.9)	
Middle Income	258 (65.8)	87 (61.3)	171 (68.4)		155 (66.2)	58 (60.4)	97 (70.3)		103 (65.2)	29 (63.0)	74 (66.1)	
High Income	53 (13.5)	16 (11.3)	37 (14.8)		32 (13.7)	13 (13.5)	19 (13.8)		21 (13.3)	3 (6.5)	18 (16.1)	
**Nativity**												
Born in U.S./Israel	376 (91.7)	141 (92.8)	235 (91.0)	0.55	227 (90.1)	97 (91.5)	130 (89.0)	0.52	149 (94.3)	44 (95.7)	105 (93.8)	0.64
**Religious status**												
**Country**												
Israel	252 (61.5)	106 (69.7)	146 (56.6)	**0.01**	-	-	-	-	-	-	-	-
U.S.	158 (38.5)	46 (30.3)	112 (43.4)		-	-	-	-	-	-	-	-
Christian	-	-	-	-	-	-	-	-	68 (44.4)	25 (56.8)	43 (39.4)	0.05
Others	-	-	-	-	-	-	-	-	85 (55.6)	19 (43.2)	66 (60.6)	
**Race/ethnicity**												
White	-	-	-	-	-	-	-	-	73 (46.2)	25 (54.3)	48 (42.9)	0.48
Black	-	-	-	-	-	-	-	-	30 (19.0)	7 (15.2)	23 (20.5)	
Asian	-	-	-	-	-	-	-	-	33 (20.9)	7 (15.2)	26 (23.2)	
Hispanic	-	-	-	-	-	-	-	-	22 (13.9)	7 (15.2)	15 (13.4)	
**Religious level**												
Religious	-	-	-	-	79 (32.8)	28 (27.5)	51 (36.7)	0.13	-	-	-	-
Not religious	-	-	-	-	162 (67.2)	74 (72.5)	88 (63.3)		-	-	-	-
**Race/ethnicity**												
Jewish	-	-	-	-	200 (79.4)	87 (82.1)	113 (77.4)	0.37	-	-	-	-
Arab	-	-	-	-	52 (20.6)	19 (17.9)	33 (22.6)		-	-	-	-

**Missing values (overall (U.S. Israel))¹**: Annual Household income *n* = 18 (0,18); Relationship status *n* = 4 (1, 3); Education level *n* = 2 (0,2); **U.S. only**: Religious status *n* = 5; **Israel only**: Religious status *n* = 11; Education level *n* = 2;

**Annual household income**: **U.S.**: Low (≤$24,999), Middle ($25,000-$149,999), and High (≥$150,000); **Israel:** Low (≤ NIS 30,000), Middle (NIS 30,001–192,000), and High (≥ 192,001 NIS); **U.S.**: United States; **SD**: Standard deviation;

**P-value: **p-values for differences across disposable versus non-disposable e-cigarette use were tested using the chi-square test for categorical variables. **P-value**^**a**^**:** Manny Whitney U test. **Bold **indicates significant at *p*<0.05

**Nativity:** for U.S., it is “born in U.S.” and for Israel, it is “born in Israel”

## Perceptions and attitudes towards E-cigarettes

Table 2 presents the perceptions and attitudes toward e-cigarettes among the total sample and stratified by country, across disposable e-cigarette use status. Those participants who used disposable e-cigarette reported a lower mean perception of the difference between e-cigarettes versus combustible cigarettes (3.3 ± 1.2) than the non-disposable group (3.7 ± 1.2) (*p* = 0.002). Mean scores for the perceptions of e-cigarette harm were notably higher among those who used disposable (5.3 ± 1.6) compared to those who used non-disposable e-cigarettes (4.8 ± 1.7) (*p* < 0.001).

The mean score for confidence in quitting e-cigarette use was significantly higher among participants who used disposable e-cigarettes (6.8 ± 3.2) compared to those who used non-disposable e-cigarettes (6.0 ± 3.2) (*p* = 0.03). There was a significant difference in the proportion of those who said they plan to quit using e-cigarettes within a month, or more than a month, between participants who use disposable e-cigarettes and those who use non-disposable e-cigarettes, but no significant difference in those reporting they had no plans to quit using e-cigarettes.

Perceptions and intentions related to e-cigarette use also varied by country and device type (Table 2). In Israel, those reporting disposable e-cigarette use perceived e-cigarettes as more harmful (5.49 ± 1.6 vs. 4.58 ± 1.7, *p* < 0.001) and expressed greater confidence in quitting (6.82 ± 1.11 vs. 5.58 ± 3.13, *p* < 0.001), compared to those reporting non-disposable e-cigarette use. No significant differences in these perceptions were observed in the U.S. group. Intentions to quit also varied: in Israel, a higher percentage of those reporting disposable use indicated plans to quit within one month (18.9% (*n* = 20) vs. 7.5% (*n* = 11), *p* < 0.001), while this pattern was not evident in the U.S.

### Use of e-cigarette, tobacco and other substances

A high proportion of participants reported current use of combustible cigarettes (68.3%, *n* = 280), alcohol (72.0%, *n* = 295), and cannabis (74.4%, *n* = 305), with no significant differences between the groups (Table 2). Almost all reported a history of combustible cigarette use (86.1%, *n* = 353), with no significant difference between the two groups (*p* = 0.80). Participants who used disposable e-cigarettes reported purchasing their products from regular shops (55.3%, *n* = 84) more frequently than those who use non-disposable e-cigarettes (33.7%, *n* = 87) (*p* < 0.001). In contrast, participants who use non-disposable e-cigarettes were more likely to purchase from online (20.2%, *n* = 52), compared to those who use disposable e-cigarettes (6.6%, *n* = 10) (*p* < 0.001). ‘Specialty’ shops were also more frequently used by those using non-disposable e-cigarettes (36.8%, *n* = 93) vs. disposable e-cigarettes (29.6%, *n* = 45) (*p* < 0.001).

Differences in substance use patterns and flavour preferences were also observed by type of e-cigarettes use and country (Table 2). In both countries, a higher proportion of individuals reporting disposable e-cigarette use reported ever using other tobacco or nicotine products, with a more pronounced difference in Israel (90.6% (*n* = 96) vs. 80.1% (*n* = 117), *p* = 0.02). Use of cannabis/cannabinoid e-liquids was more frequently reported by those using non-disposable products in both Israel (56.8% (*n* = 83) vs. 29.2% (*n* = 31), *p* < 0.001) and the U.S. (45.5% (*n* = 51) vs. 28.3% (*n* = 13), *p* = 0.04). Among U.S. participants, sweet-flavoured e-cigarettes were more commonly reported among those using disposable products compared to non-disposable ones (84.8% (*n* = 39) vs. 53.6% (*n* = 60), *p* < 0.001), a pattern not observed in Israel. Menthol/mint flavour use was more frequent among U.S. individuals reporting non-disposable use (46.4% (*n* = 52) vs. 28.3% (*n* = 13), *p* = 0.04), while rates were comparable across product types in Israel. In terms of purchase locations, ‘regular’ shops were the most frequently reported source in Israel (43.7%, *n* = 110), particularly among those using disposable (60.4%, *n* = 64) vs. non-disposable e-cigarettes (31.5%, *n* = 46). In contrast, in the United States, ‘specialty’ shops were the most common purchase location overall (43.7%, *n* = 69), with usage rates being relatively similar between those using disposables (41.3%, *n* = 19) vs. non-disposables (44.6%, *n* = 50).

**Table 2:** Perceptions and attitudes towards e-cigarettes and use characteristics across the total sample, and stratified by country, across disposable versus non-disposable e-cigarette use (*n* = 410)

	Overall (*n* = 410)				Israel (*n* = 252)				U.S. (*n* = 158)			
Variables	**Total** **(n = 410) **	**Disposable** **(n = 152)**	**Non-disposable** **(n = 258) **	**p-value**	**Total** **(n = 252)**	**Disposable** **(n = 106)**	**Non-disposable** **(n = 146) **	**p-value**	**Total** **(n = 158) **	**Disposable** **(n = 46)**	**Non-disposable** **(n = 112) **	**p-value**
**Perceived difference between e-cigarettes & cigarettes**	3.6 ± 1.17	3.3 ± 1.2	3.7 ± 1.2	**0.002** ^**a**^	3.31 ± 1.16	3.13 ± 1.147	3.45 ± 1.16	**0.03** ^**a**^	4.0 ± 1.1	3.8 ± 1.1	4.1 ± 1.0	0.15ᵃ
**Perception of e-cigarette information**	2.8 ± 1.1	3.0 ± 1.1	2.8 ± 1.1	0.08ᵃ	2.72 ± 1.04	2.91 ± 1.06	2.58 ± 1.01	**0.02** ^**a**^	3.0 ± 1.2	3.1 ± 1.2	3.0 ± 1.2	0.75ᵃ
**Perceived e-cigarette addictiveness**	5.0 ± 1.9	5.1 ± 1.9	4.9 ± 1.9	0.24ᵃ	4.83 ± 1.91	5.08 ± 1.8	4.66 ± 1.9	0.08ᵃ	5.3 ± 1.8	5.3 ± 1.8	5.3 ± 1.9	0.85ᵃ
**Perceived e-cigarette harm**	5.0 ± 1.7	5.3 ± 1.6	4.8 ± 1.7	**< 0.001** ^**a**^	4.96 ± 1.71	5.49 ± 1.6	4.58 ± 1.7	**< 0.001** ^**a**^	5.0 ± 1.6	5.0 ± 1.5	5.0 ± 1.6	0.89ᵃ
**Heath warning label effect**												
Didn’t notice	37 (9.0)	13 (8.6)	24 (9.4)	0.95	27 (10.8)	10 (9.4)	17 (11.7)	0.43	10 (6.4)	3 (6.5)	7 (6.3)	0.76
Concerned	96 (23.5)	34 (22.4)	62 (24.2)		46 (18.3)	19 (17.9)	27 (18.6)		50 (31.8)	15 (32.6)	35 (31.5)	
Reassured	112 (27.5)	42 (27.6)	70 (27.3)		88 (35.1)	33 (31.1)	55 (37.9)		24 (15.3)	9 (19.6)	15 (13.5)	
Had no effect	163 (40.0)	63 (41.4)	100 (39.1)		90 (35.9)	44 (41.5)	46 (31.7)		73 (46.5)	19 (41.3)	54 (48.6)	
**Importance of quitting e-cigarette use**	5.6 ± 3.3	5.4 ± 3.4	5.7 ± 3.3	0.32ᵃ	5.88 ± 3.22	5.71 ± 3.32	6.00 ± 3.14	0.57ᵃ	5.1 ± 3.5	4.5 ± 3.5	5.4 ± 3.5	0.18ᵃ
**Confidence in quitting e-cigarette use¹**	6.3 ± 3.2	6.8 ± 3.16	6.03 ± 3.2	**0.03** ^**a**^	6.10 ± 3.18	6.82 ± 3.11	5.58 ± 3.13	**< 0.001** ^**a**^	6.6 ± 3.2	6.6 ± 3.3	6.6 ± 3.2	0.94ᵃ
**E-cigarettes quit attempts in the last year**												
Attempted to quit	249 (60.7)	91 (59.9)	158 (61.2)	0.78	176 (69.8)	70 (66.0)	106 (72.6)	0.26	73 (46.2)	21 (45.7)	52 (46.4)	0.93
Didn’t attempt to quit	161 (39.3)	61 (40.1)	100 (38.8)		76 (30.2)	36 (34.0)	40 (27.4)		85 (53.8)	25 (54.3)	60 (53.6)	
**Plan to quit e-cigarettes**												
≤ 1month	51 (12.4)	26 (17.1)	25 (9.7)	**0.002**	31 (12.3)	20 (18.9)	11 (7.5)	**< 0.001**	20 (12.7)	6 (13.0)	14 (12.5)	0.53
> 1month	179 (43.7)	50 (32.9)	129 (50.0)		117 (46.4)	35 (33.0)	82 (56.2)		62 (39.2)	15 (32.6)	47 (42.0)	
No plan to quit	180 (43.9)	76 (50.0)	104 (40.3)		104 (41.3)	51 (48.1)	53 (36.3)		76 (48.1)	25 (54.3)	51 (45.5)	
**Likelihood of continued e-cigarette use**	4.4 ± 2.2	4.2 ± 2.1	4.5 ± 2.2	0.11ᵃ	4.20 ± 1.16	3.92 ± 2.06	4.41 ± 2.07	**0.05** ^**a**^	4.6 ± 2.3	4.7 ± 2.2	4.6 ± 2.3	0.84ᵃ
**Ever-combustible cigarettes use**	353 (86.1)	130 (85.5)	223 (86.4)	0.80	216 (85.7)	89 (84.0)	127 (87.0)	0.50	137 (86.7)	41 (89.1)	96 (85.7)	0.57
Ever use of other tobacco or nicotine products*	348 (84.9)	140 (92.1)	208 (80.6)	**0.002**	213 (84.5)	96 (90.6)	117 (80.1)	**0.02**	135 (85.4)	44 (95.7)	91 (81.3)	**0.02**
**Current combustible cigarette use**	280 (68.3)	105 (69.1)	175 (67.8)	0.79	198 (78.6)	81 (76.4)	117 (80.1)	0.48	82 (51.9)	24 (52.2)	58 (51.8)	0.97
**Current alcohol use**	295 (72.0)	102 (67.1)	193 (74.8)	0.09	176 (69.8)	68 (64.2)	108 (74.0)	0.09	119 (75.3)	34 (73.9)	85 (75.9)	0.79
**Current cannabis use**	305 (74.4)	121 (79.6)	184 (71.3)	0.06	204 (81.0)	89 (84.0)	115 (78.8)	0.30	101 (63.9)	32 (69.6)	69 (61.6)	0.34
**Current use of other tobacco or nicotine products**	356 (86.8)	137 (90.1)	219 (84.9)	0.13	211 (83.7)	95 (89.6)	116 (79.5)	**0.03**	145 (91.8)	42 (91.3)	103 (92.0)	0.89
**Daily e-cigarette puff use¹**												
0–39	316 (77.3)	114 (75.0)	202 (78.6)	0.40	207 (82.1)	83 (78.3)	124 (84.9)	0.18	109 (69.4)	31 (67.4)	78 (70.3)	0.72
≥ 40	93 (22.7)	38 (25.0)	55 (21.4)		45 (17.9)	23 (21.7)	22 (15.1)		48 (30.6)	15 (32.6)	33 (29.7)	
**Daily use of e-cigarettes for the past 30 days¹**	204 (50.1)	84 (55.3)	120 (47.1)	0.11	136 (54.0)	61 (57.5)	75 (51.4)	0.33	68 (43.9)	23 (50.0)	45 (41.3)	0.32
**Cannabis/cannabinoids e-liquid use**	178 (43.4)	44 (28.9)	134 (51.9)	**< 0.001**	114 (45.2)	31 (29.2)	83 (56.8)	**< 0.001**	64 (40.5)	13 (28.3)	51 (45.5)	**0.04**
**Nicotine Concentration**												
< 5%(< 50 mg/ml)	239 (58.3)	79 (52.0)	160 (62.0)	0.05	167 (66.3)	63 (59.4)	104 (71.2)	0.05	72 (45.6)	16 (34.8)	56 (50.0)	0.08
≥ 5%(≥ 50 mg/ml)	171 (41.7)	73 (48.0)	98 (38.0)		85 (33.7)	43 (40.6)	42 (28.8)		86 (54.4)	30 (65.2)	56 (50.0)	
**E-cigarettes purchase locations**												
Online Shops	62 (15.1)	10 (6.6)	52 (20.2)	**< 0.001**	49 (19.4)	7 (5.5)	42 (28.8)	**< 0.001**	13 (8.2)	3 (6.5)	10 (8.9)	0.86
Speciality Shops	138 (33.7)	45 (29.6)	93 (36.8)		69 (27.4)	26 (24.5)	43 (29.5)		69 (43.7)	19 (41.3)	50 (44.6)	
Regular shopsᵇ	171 (41.7)	84 (55.3)	87 (33.7)		110 (43.7)	64 (60.4)	46 (31.5)		61 (38.6)	20 (43.5)	41 (36.6)	
Others	39 (9.5)	13 (8.6)	26 (10.1)		24 (9.5)	9 (8.5)	15 (10.3)		15 (9.5)	4 (8.7)	11 (9.8)	
**Tobacco-flavoured e-cigarette use**	84 (20.5)	23 (15.1)	61 (23.6)	**0.04**	54 (21.4)	16 (15.1)	38 (26.0)	**0.04**	30 (19.0)	7 (15.2)	23 (20.5)	0.44
**Menthol or mint-flavoured e-cigarette use**	141 (34.4)	45 (29.6)	96 (37.2)	0.12	76 (30.2)	32 (30.2)	44 (30.1)	0.99	65 (41.1)	13 (28.3)	52 (46.4)	**0.04**
**Sweet-flavoured (Candy, caramel, fruit) e-cigarette use**	267 (65.1)	112 (73.7)	155 (60.1)	**0.005**	168 (66.7)	95 (65.1)	95 (65.1)	0.53	99 (62.7)	39 (84.8)	60 (53.6)	**< 0.001**
**Others (alcohol, coffee or tea, other foods) use**	84 (20.5)	36 (23.7)	48 (18.6)	0.22	62 (24.6)	26 (24.5)	36 (24.7)	0.98	22 (13.9)	10 (21.7)	12 (10.7)	0.07
**E-cigarette use upon waking in the morning**				0.69								
≤ 5 min	61 (14.9)	24 (15.8)	37 (14.3)		21(8.3)	9(8.5)	12 (8.2)	0.94	40 (25.3)	15 (32.6)	25 (22.3)	0.18
> 5 min	349 (85.1)	128 (84.2)	221 (85.7)		231(91.7)	97(91.5)	134(91.8)		118 (74.7)	31 (67.4)	87 (77.7)	

**Missing value¹: **Perception of e-cigarette information n = 24; Perceived e-cigarette addictiveness n=6; Perception of harm of e-cigarettes n=3; Effect of health warnings have on your thoughts about using e-cigarettes n=2; Confidence in quitting e-cigarette use n=3; How likely are you to try or continue to use e-cigarettes in the next year n= 5

**Missing value in U.S. data only:** Daily e-cigarette puff use n= 1; Daily use of e-cigarettes for the past 30 days n=3

**p-value**^**a**^**:** p-values for differences across disposable versus non-disposable e-cigarette use were tested using the Manny-Whitney U test for ordinary categorical variables. Other p-values are the result of the chi-square test. **U.S.:** United States; **SD:** Standard deviation;** Bold** indicates significance at p<0.05.**p-value**^**a**^**:** p-values for differences across disposable versus non-disposable e-cigarette use were tested using the Manny-Whitney U test. Other p-values are the result of the chi-square test

**Scale: Perception of harm: **1= Not at all harmful to 7=Extremely harmful; **Perceived addictiveness:** 1= Not at all addictive to 7=Extremely addictive; **Perception of the difference between e-cigarettes and combustible cigarettes:** Not at all (1), A little (2), Neutral/unsure (3), Somewhat (4), Very (5); **Perception about e-cigarette information:** Mostly positive(1), Slightly positive(2), Equally balanced(3), Slightly negative(4), Mostly negative(5); **Importance of quitting e-cigarette use:** 0-10: 0 being not at all important and 10 being absolutely important; **Confidence in quitting e-cigarette use:** 0-10: 0 being not at all confident and 10 being absolutely confident; **How likely are you to try or continue to use e-cigarettes in the next year:** 1=Not at all likely to 7=Extremely likely

*Other tobacco or nicotine products: Heated tobacco products, Hookah, Waterpipe, or Nargila, Large or little cigars, Pipe with tobacco, Smokeless tobacco. **Regular shops**^**a**^ = Gas stations, convenience stores, grocery stores, pharmacy, a small kiosk; **Sweet flavoured** = Candy flavors (for example, licorice, gummy bears, or bubble gum), caramel, vanilla, chocolate, cream, fruit flavour

### Multivariable logistic Regression, stratified by country

Table 3 presents the adjusted odds ratios (aOR) for disposable e-cigarette use by socio-demographic characteristics, use characteristics, and perception factors, stratified by country (Israel and the U.S.).

### Israel

In Israel, there was no significant association between the use of disposable e-cigarettes among 18–25 years old (aOR 1.66, 95%CI: 0.68–4.05) and 26–35 years old (aOR 1.41, 95%CI: 0.63–3.16) compared to 36–45 years old. Those participants with a higher perception of e-cigarette health harms had higher odds of using disposable e-cigarettes (aOR 1.45, 95%CI: 1.20–1.76) than non-disposable e-cigarettes. Those who used cannabis/cannabinoid-containing e-liquids were significantly less likely to use disposable e-cigarettes (aOR 0.36, 95%CI:0.19–0.69), compared to those who did not use these substances. Compared to those purchasing e-cigarettes online, those buying from ‘regular’ shops or from ‘specialty’ shops showed a higher likelihood of using disposable e-cigarettes (aOR 6.30, 95%CI: 2.25–17.67; and aOR 3.71, 95%CI: 1.21–11.34), respectively. However, when comparing ‘regular’ shops and ‘specialty’ shops, there was no statistically significant difference in disposable e-cigarette use between these two groups (aOR 0.59, 95% CI: 0.29–1.22, *p* = 0.15).

### United States

In the U.S., ever-use of other tobacco or nicotine products was significantly associated with disposable e-cigarette use (aOR 7.51, 95%CI: 1.49–37.87), compared to those who had never used other products. Preference for sweet flavors was significantly associated with disposable e-cigarette use (aOR 4.42, 95%CI: 1.64–11.94), compared to non-disposable e-cigarette use. Those who used cannabis/cannabinoid-containing e-liquids were less likely to use disposable e-cigarettes (aOR 0.33, 95%CI: 0.13–0.82), compared to those who did not use cannabis/cannabinoid-containing e-liquids. Individuals in the 18–25 age group did not have a statistically significant increased likelihood of using disposable e-cigarettes compared to those in the 36–45 age group (aOR 1.40, 95%CI: 0.49–4.03, *p* = 0.53), while individuals in the 26–35 age group were significantly less likely to use disposable e-cigarettes compared to those in the 36–45 age group (aOR 0.36, 95%CI: 0.13–0.98, *p* = 0.04).

**Table 3:** Multivariable logistic regression for the association between demographics, perception factors, use characteristics, And disposable e-cigarette use among adults in U.S. And Israel

	Israel (*n* = 252)				U.S. (*n* = 158)			
	**Total (n = 252)** *n* (%)	Adjusted OR	95% CI	*p*-value	**Total (n = 158)** *n* (%)	Adjusted OR	95% CI	*p*-value
**Age (Ref: 36–45)**								
18–25	91 (36.1)	1.66	0.68–4.05	0.26	39 (24.7)	1.40	0.49–4.03	0.53
26–35	89 (35.3)	1.41	0.63–3.16	0.41	60 (38.0)	0.36	0.13–0.98	**0.04**
**Gender (Ref: Male)**								
Female	112 (44.4)	1.32	0.68–2.55	0.41	71 (44.9)	1.02	0.44–2.36	0.97
**Employment status (Ref: Employed)**								
Unemployed	69 (27.4)	1.02	0.47–2.23	0.96	46 (29.1)	0.79	0.31–1.99	0.79
**Relationship status (Ref: Married/cohabitating)**								
Single/Divorced/Widowed	97 (39.0)	1.76	0.85–3.65	0.18	80 (51.0)	1.61	0.67–3.85	0.28
**Perceived difference between e-cigarettes & combustible cigarettes (mean ± SD)**	3.3 ± 1.2	0.77	0.60–1.01	0.06	4.0 ± 1.1	0.74	0.49–1.10	0.14
**Perceived e-cigarette harm (mean ± SD)**	5.0 ± 1.7	1.45	1.20–1.76	**< 0.001**	5.0 ± 1.7	1.04	0.80–1.34	0.79
**Plan to quit e-cigarettes (Ref: No plan to quit)**								
≤ 1month	31 (12.3)	1.62	0.61–4.33	0.34	20 (12.7)	0.83	0.23–2.96	0.78
>1month	117 (46.4)	0.57	0.28–1.15	0.12	62 (39.2)	0.57	0.23–1.45	0.24
Ever use of other tobacco or nicotine products* (Ref: No)								
Yes	213 (84.5)	0.81	0.28–2.31	0.67	135 (85.4)	7.51	1.49–37.87	**0.02**
**Cannabis/cannabinoid e-liquid use (Ref: No)**								
Yes	114 (45.2)	0.36	0.19–0.69	**0.002**	64 (40.5)	0.33	0.13–0.82	**0.02**
**E-cigarette purchase locations (Ref: Online shops)**								
Speciality Shops	69 (27.4)	3.71	1.21–11.34	**0.02**	69 (43.7)	1.11	0.22–5.69	0.90
Regular shopsᵇ	110 (43.7)	6.30	2.25–17.67	**< 0.001**	61 (38.6)	1.50	0.29–7.70	0.63
Others	24 (9.5)	2.60	0.66–10.15	0.17	15 (9.5)	1.00	0.13–8.01	0.99
**Sweet flavoured (fruit, candy, caramel) e-cigarette use**								
Yes (Ref: No)	168 (66.7)	1.51	0.77–2.97	0.23	99 (62.7)	4.42	1.64–11.94	**0.003**

*Other tobacco or nicotine products: Heated tobacco products, Hookah, Water-pipe, or Nargila, Large or little cigars, Pipe with tobacco, Smokeless tobacco

**Regular shops**^**a**^ = Gas stations, convenience stores, grocery stores, pharmacy, a small kiosk. **Bold** indicates significant at *p* < 0.05; **SD**: Standard deviation. **Scale: Perception of harm**: 1 = Not at all harmful to 7 = Extremely harmful; **Perception of the difference between e-cigarettes and combustible cigarettes**: 1= Not at all, 2=A little, 3=Neutral/unsure, 4=Somewhat, 5=Very

Pairwise comparison between Regular shops vs. Specialty shops (aOR0.59, 95% CI: 0.29–1.22, p = 0.15)

## Discussion

This study provides a detailed analysis of the sociodemographic characteristics, perceptions, attitudes, and usage patterns of those who reported using disposable e-cigarettes versus those who reported using non-disposable e-cigarettes among adults in Israel and the U.S. Our findings highlight significant socio-demographic and behavioral differences between the two groups. In both countries, the use of cannabis/cannabinoid-containing e-liquids was a key factor associated with using non-disposable e-cigarettes. In the U.S., disposable e-cigarette use was linked to a preference for sweet flavors, ever use of other tobacco or nicotine products, and older age groups among adults. In Israel, disposable e-cigarette use was associated with a higher perception of e-cigarette harm and purchasing products from ‘regular’ shops and ‘specialty’ shops compared to ‘online’ shops.

Disposable e-cigarette use (vs. non-disposable use) was more prevalent in Israel than in the U.S. This may reflect regional differences in market availability, cultural acceptance, and regulatory policies affecting e-cigarette accessibility [34]. Notably, 14.5% of adult who used disposable e-cigarettes had never smoked combustible cigarettes, emphasizing the appeal of e-cigarettes as a standalone product rather than just as a harm reduction tool or smoking cessation aid. A previous U.S. study among young adults showed that individuals who had never smoked combustible cigarettes were more likely to initiate disposable e-cigarette use, highlighting a concerning trend of nicotine uptake among those who had never smoked [12]. Nonetheless, in the U.S. sample, participants who had ever used other tobacco or nicotine products (i.e., regardless of combustible cigarette use) were seven times more likely to use disposable e-cigarettes. Our results align with previous studies that have found a strong association between disposable e-cigarette use and prior tobacco or nicotine product use [12]. Even though there was no significant difference in the history of combustible cigarette use between the groups, those using disposables were more likely to have used other tobacco or nicotine products. This could suggest that disposable e-cigarettes serve as an introductory product for exploring alternative nicotine delivery systems among people who smoke [12, 35]. Determining whether disposable e-cigarettes serve as a gateway to nicotine experimentation requires longitudinal research that follows individuals with no prior tobacco or nicotine use to track initiation sequences and transitions over time.

Surprisingly, in our sample younger age was not associated with using disposable e-cigarette use, which is in contrast to the literature [12, 18, 28, 31]. Previous studies suggest that younger individuals are more inclined to use disposable e-cigarettes due to their convenience and lower initial cost [28, 36]. Another reason reported for the preference for disposable e-cigarettes among young adults is their dissatisfaction with the high costs and times required to purchase and upgrade rechargeable devices and refill e-cigarette solutions [37]. Despite younger age [18–24] being associated with disposable e-cigarette use in the bivariate analysis of the Israel data, after adjustment in the regression analysis, age maintained a similar trend but was no longer significantly associated with disposable e-cigarette use. This could be attributed to the limitations of our sample, which may not be representative of the general adult smoking population [18], as well as the small overall sample size within each age group. It is possible that residual confounding may also be present. However, it should be noted that most studies focused on youth (under 18 years old), which were not included in this sample [19]. To address these gaps, future studies should oversample younger adults and include adolescents to capture the full age spectrum of disposable device uptake, thereby enabling clearer age-related inference.

For the Israeli sample, our findings suggest that perceived harm might be a more significant factor than age in influencing the use of disposable versus non-disposable e-cigarettes among adults. Israeli participants who used disposable e-cigarettes had higher perceptions of e-cigarette harms (i.e., viewed e-cigarettes as more harmful) compared to those who used non-disposable e-cigarettes. This may be because they view disposable e-cigarettes as less harmful than non-disposable e-cigarettes and combustible cigarettes [31, 38]. This paradox suggests cognitive dissonance, where those people who used disposable e-cigarettes acknowledge the risks but underestimate their addiction potential [31]. Additionally, disposables might be perceived as a one-time purchase, signaling less consistent use compared to non-disposables, and therefore less harmful [31]. Given these complex and sometimes contradictory perceptions, further qualitative research is needed to explore the psychological mechanisms underlying harm perceptions, such as risk minimization, cognitive dissonance, and device-specific beliefs, and how these processes shape device choice.

In Israel, disposable e-cigarette use was significantly associated with purchasing in ‘regular’ shops and ‘specialty’ shops compared to ‘online’ shops. Aligned with our findings, a previous 2022 Israeli study revealed that 42% of adolescents purchased smoking products from grocery stores and kiosks, followed by supermarkets (15.9%), tobacco specialty stores (14.3%), and convenience stores at gas stations (14.3%) [19]. The unsupervised sale of tobacco and nicotine products at these locations, despite legal restrictions, might have contributed to rising rates of use among adolescents due to the widespread availability of these products and lack of enforcement [19]. The tendency for those participants who used disposable e-cigarettes to purchase from ‘regular’ shops and ‘specialty’ shops rather than ‘online’ shops may indicate ease of accessibility and convenience in terms of physical locations as significant factors in product purchase place choice [31], and can serve as important considerations for policy. Building on these observations, future research should evaluate how retail density, in-store marketing, and enforcement levels influence product choice, particularly among populations with limited online access or higher retail exposure.

In both countries, those who used disposable e-cigarettes were less likely to use cannabis/cannabinoid-containing e-liquids. This is probably explained by the fact that disposable e-cigarettes come with prefilled e-liquid components with no capacity to change the liquid [1, 5]. The positive association between non-disposable e-cigarettes and cannabis/cannabinoid-containing e-liquid use both in the U.S. and Israel might be explained by the high level of concomitant use of e-cigarettes and cannabis among those who use e-cigarettes [39]. A study conducted in Canada found that e-cigarette use among individuals aged 15–24 was significantly associated with cannabis use, with those using cannabis being significantly more likely to use e-cigarettes compared to those who don’t use e-cigarettes [39]. Importantly, it is also relevant to note that most documented cases of serious harm from e-cigarettes such as EVALI (e-cigarette or vaping product use–associated lung injury) were linked to components found specifically in cannabis/cannabinoid-containing e-liquids [4, 40]. These patterns highlight the importance of device modifiability in shaping co-use behaviors, underscoring the need for future toxicological and behavioral studies to examine how device modifiability interacts with cannabis use, particularly in regulatory environments where cannabis products vary in legality and formulation.

### Policy implications and recommendations

Current findings, alongside what is already known from other research, provide a strong basis for several policy recommendations. Our findings highlight the need for nuanced, targeted regulation of e-cigarettes that considers the diverse patterns of use across Israel and U.S. adults. The widespread use of disposable e-cigarettes among adults and their rising popularity among youth suggest the need for balanced regulation for competing public health priorities. On one hand, stricter regulation or bans on disposable e-cigarettes may help reduce initiation and continued use among youth particularly those who have never smoked combustible cigarettes. On the other hand, overly broad restrictions could unintentionally undermine harm-reduction strategies for adults who currently smoke and use disposable e-cigarettes as a less harmful alternative.

A more effective policy approach may require differentiated strategies for distinct e-cigarette user groups. Policies aimed at curbing youth uptake could include a ban on flavored disposable e-cigarettes, stronger age-verification enforcement, and tighter marketing restrictions that reduce youth appeal. In contrast, for adults who smoke and use disposable e-cigarettes as harm reduction, regulations could focus on ensuring product safety, and maintaining availability of tobacco-flavored options through licensed channels. For example, Australia has recently adopted a policy banning the sales of e-cigarettes except via pharmacies to adults who smoke, with a prescription needed in certain circumstances such as for e-cigarettes containing a high (over 20 mg/ml) nicotine dose [41].

In addition, specifically for Israel, public education needs to clarify misconceptions about the relative risks of different e-cigarette products, as perception of e-cigarettes harm was associated with disposable e-cigarettes use in Israel.

Notably, in our survey, 13.9% of all participants, and 14.5% of those using disposable e-cigarettes had never smoked combustible cigarettes. This is concerning, as e-cigarettes will only offer a net public health benefit if they primarily appeal to adults who smoke cigarettes, without attracting use among those who have never smoked. A recent UK study showed that in the last decade there was also a concerning increase of e-cigarette use among people who have quit smoking successfully in the past before e-cigarettes were available (i.e., relapsed to nicotine addiction through using e-cigarettes) [42]. These data suggest that tighter regulation on e-cigarettes is needed to protect people who have never smoked and those who have quit smoking [42]. Specifically in the US, stronger restrictions on e-cigarette marketing are needed, such as those already implemented in Israel, including a point-of-sale display ban, plain packaging, and a complete advertisement ban [24].

Several countries have banned flavors for e-cigarettes [43, 44]. In Israel, there is currently a bill to ban flavors in all tobacco and nicotine products that is under consideration with the Ministry of Health [45]. In the U.S., the FDA has not provided authorization for multiple e-cigarette products that are flavored, and has only approved marketing of tobacco flavored e-cigarettes [46]. Despite this, currently the U.S. market does contain numerous flavored products [47]. Finding from this study suggest that banning sweet flavored e-cigarette products might also aid in reducing disposable e-cigarette use, specifically in the U.S. However, enforcement of such bans should also address synthetic flavor analogues and additives that mimic sensory effects of flavors, as these have been used to circumvent existing flavor restrictions [48].

The high level of use of cannabis/cannabinoid-containing e-liquid suggests that stricter regulation and enforcement is needed on non-disposable e-cigarette devices and e-liquids in both countries. In countries where cannabinoid e-liquids are illegal (such as in Israel), having license schemes for selling tobacco and nicotine products could help reduce the use of non-legal e-liquid and/or device manipulations.

Importantly, as new policies are adopted, rigorous and continuous evaluation will be essential to determine whether they effectively reduce harms among youth and non-smokers while preserving harm reduction benefits for adults who smoke.

### Strengths and limitations

This study contributes to an under-researched area by assessing the factors associated with the use of disposable versus non-disposable e-cigarettes in two different countries. Its cross-sectional design allowed for the examination of multiple variables and provided a cost-effective snapshot of the phenomenon. However, this nature also limits the ability to infer causal relationships, and the reliance on self-reported data introduces potential bias due to social desirability. Despite these limitations, the comparison of data from the two countries enhanced the validity of the findings. Nevertheless, the relatively small sample size may limit the generalizability of the results, particularly when analysing variables with low frequency, as manifested by wide confidence intervals, reducing the precision of the study findings.

### Future research directions

Future studies should focus on longitudinal data to understand the temporal dynamics of e-cigarette use and perceptions, particularly as individuals transition between combustible products, disposable e-cigarettes, and non-disposable e-cigarettes. Broader and more diverse samples are needed to capture variations in e-cigarette use patterns across different cultural and socioeconomic contexts. Additionally, research on the impact of regulatory changes on e-cigarette use, as well as qualitative investigations into use motivations and experiences, will provide valuable insights for shaping public health interventions and policy.

## Conclusion

This study underscores critical differences between those who used disposable e-cigarettes and those who used non-disposable e-cigarettes in the U.S. and Israel, highlighting the importance of tailored public health strategies that cater to the unique characteristics and needs of these groups. Findings suggest that various policy changes need to be considered, targeted to different population groups, such as a ban on flavors, stronger age-verification enforcement, and tighter marketing restrictions. Implementing a license scheme and better enforcement could be implemented to reduce use of illegal e-liquids.

## Supplementary Information

Supplementary Material 1.

## Data Availability

The data presented in this study are available on request from the corresponding author. The data are not publicly available due to ethical reasons.

## Abbreviations

FDA: Food and Drug Administration
CDC: Center for Disease Control
U.S.: United States
UK: United Kingdom
SD: Standard Deviation
aOR: adjusted Odds Ratio
COR: Crude Odds Ratio
CI: Confidence Interval
KP: Knowledge Panel
CBD: Cannabinoids
