# Supplementary figures and images for: Factors associated with using disposable versus non-disposable electronic cigarettes among adults in the U.S. and Israel: a cross-sectional study with policy implications
Source: Isr J Health Policy Res. 2025 Dec 3;14:73. doi: 10.1186/s13584-025-00738-9 (PMC12673750; doi:10.1186/s13584-025-00738-9)

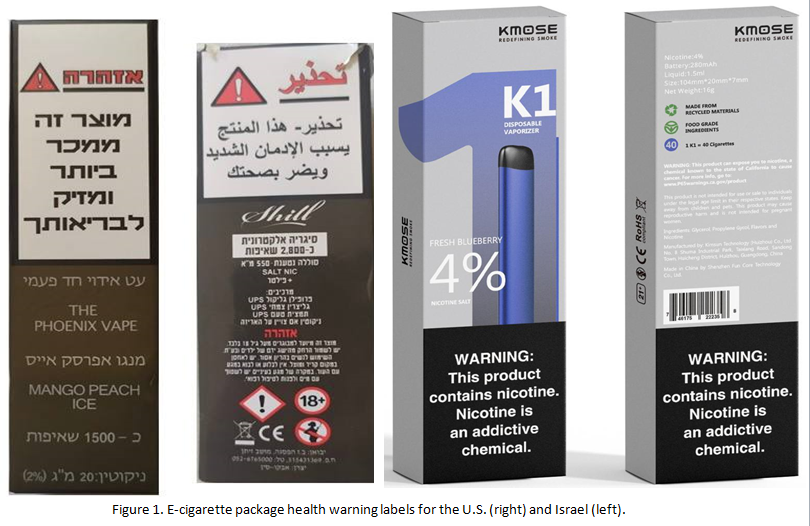

Supplement: Supplementary file 1 — Supplementary Material 1. [file 13584_2025_738_MOESM1_ESM.png]
